# Network Pharmacology Approach to Uncover the Mechanism Governing the Effect of Simiao Powder on Knee Osteoarthritis

**DOI:** 10.1155/2020/6971503

**Published:** 2020-12-07

**Authors:** Zhengquan Huang, Xiaoqing Shi, Xiaochen Li, Li Zhang, Peng Wu, Jun Mao, Runlin Xing, Nongshan Zhang, Peimin Wang

**Affiliations:** Department of Orthopaedics and Traumatology, The First Affiliated Hospital of Nanjing University of Chinese Medicine, Jiangsu Provincial Hospital of Traditional Chinese Medicine, Nanjing, China

## Abstract

**Objective:**

To explore the molecular mechanism of Simiao powder in the treatment of knee osteoarthritis.

**Methods:**

Based on oral bioavailability and drug-likeness, the main active components of Simiao powder were screened using the Traditional Chinese Medicine Systems Pharmacology Database and Analysis Platform (TCMSP). GeneCard, OMIM, DisGeNET, DrugBank, PharmGkb, and the Therapeutic Target Database were used to establish target databases for knee osteoarthritis. Cytoscape software was used to construct a visual interactive network diagram of “active ingredient - action target – disease.” The STRING database was used to construct a protein interaction network and analyze related protein interaction relationships. Kyoto Encyclopedia of Genes and Genomes (KEGG) pathway and Gene Ontology (GO) biological process enrichment analysis were performed on the core targets. Additionally, Discovery Studio software was used for molecular docking verification of active pharmaceutical ingredients and disease targets.

**Results:**

Thirty-seven active components of Simiao powder were screened, including 106 common targets. The results of network analysis showed that the targets were mainly involved in regulating biological processes such as cell metabolism and apoptosis. Simiao powder components were predicted to exert their therapeutic effect on the AGE-RAGE signaling pathway in diabetic complications, IL-17 signaling pathway, TNF signaling pathway, Toll-like receptor signaling pathway, and HIF-1 signaling pathway. The molecular docking results showed that the active components of Simiao powder had a good match with the targets of IL1B, MMP9, CXCL8, MAPK8, JUN, IL6, MAPK1, EGF, VEGFA, AKT1, and PTGS2.

**Conclusion:**

Simiao powder has multisystem, multicomponent, and multitarget characteristics in treating knee osteoarthritis. Its possible mechanism of action includes inhibiting the inflammatory response, regulating immune function, and resisting oxidative stress to control the occurrence and development of the disease. Quercetin, wogonin, kaempferol, beta-sitosterol, and other active ingredients may be the material basis for the treatment of knee osteoarthritis.

## 1. Introduction

Knee osteoarthritis (KOA) is a common disease in middle-aged and elderly populations. The main clinical manifestations are knee pain, stiffness, and limited joint activity, and severe patients may even have disability [1]. KOA has widespread distribution in the world, is the main cause of disability, and has a negative impact on physical and mental health [1]. With the rapid growth of the global aging population, KOA has gradually becomes a major health problem affecting physical and mental health [2]. Chronic pain is the most common symptom of KOA. Relieving pain symptoms and improving quality of life in patients with KOA are urgent needs [3].

Chinese herbal medicines (CHMs) have been used in China for thousands of years. Simiao powder use in treatment of KOA was first recorded in the book Cheng Fang Bian Du written by Bingcheng Zhang in the Qing Dynasty of China. Simiao powder is composed of four herbs: *Atractylodes chinensis rhizome* (*Rhizoma aractylodis*), *Cortex phellodendri*, *Radix achyranthis bidentatae*, and *Coicis Semen* (*Coix seed*).

The rhizome of *Atractylodes chinensis rhizome* is derived from the dry rhizome of the compositae *Atractylodes lancea* (*Thunb.*) *DC* or *Atractylodes chinensis* (*DC.*) *Koidz*. It eliminates dampness, strengthens the spleen, eliminates wind, and disperses cold. Modern pharmacological studies have found that the main active components of *Atractylodes chinensis rhizome* include atractylenolide I, atractylenolide II, atractylenolide III, atractylone, hinesol, *β*-eudesmol, atractylodin, stigmasterol, and *β*-sitosterol [4]. Modern studies have confirmed the anti-inflammatory effects of atractylenolide I (ATL-1) [5, 6]. ATL-1 has been shown to inhibit angiogenesis by downregulating NO, TNF-*α*, IL-1*β*, IL-6, VEGF, and PlGF in chronic inflammation [5]. At the same time, ATL-1 inhibits increased blood vessel permeability caused by acetic acid in mice and swelling of the foot plantar caused by carrageenan in mice [7]. Additionally, *Atractylodes chinensis rhizome* has antitumor effects. Bladder cancer is a critical urological disease. Modern studies have confirmed that ATL-1 has therapeutic effects on bladder cancer in vivo and in vitro by increasing the level of P21 and decreasing the levels of cyclin B1, CDK1, and Cdc25c [8]. Melanoma is a type of skin cancer. ATL-1 was shown to inhibit the growth of human melanoma cells by inducing apoptosis [9]. Notably, some of the active ingredients of *Atractylodes chinensis rhizome* are also effective against leukemia and allergic diseases [10].

Cortex phellodendri is the dry bark of the rutaceae plants *Phellodendron chinense Schneid* or *Phellodendron amurense Rupr*. It contains many active components, such as alkaloid, sterol, lactone, and mucinous substance [11]. Cortex phellodendri and its extracts have anti-inflammatory, antibacterial, immunosuppressive, antiulcer, and other effects [12]. Alkaloid is an important active substance in Cortex phellodendri, and berberine is the most abundant component. Berberine inhibits sodium nitroprusside by activating AMPK signaling and inhibiting p38 MAPK activity, thereby inhibiting chondrocyte apoptosis stimulated by sodium nitroprusside and improving cartilage degeneration [13]. Other studies have shown that berberine can improve the degeneration of osteoarthritis (OA) cartilage by stimulating activation of articular chondrocytes and AKT in the rat OA model with IL-1 stimulation, thereby promoting survival of chondrocytes and stromal production [14].

The source of Radix Achyranthis bidentatae is the root and rhizome of Achyranthes aspera L. The main active ingredients of Radix Achyranthis bidentatae include saponins, sterones, flavonoids, sugar, alkaloids, and organic acids. Among them, saponins and sterones are the main chemical components of Radix Achyranthis bidentatae [15]. Previous studies reported that the possible mechanisms of action of RAB include regulating immune and inflammatory responses, reducing chondrocyte apoptosis, and protecting joint synovium and cartilage to control disease development [16]. Saponins of Achyranthes bidentata have protective effects on interleukin-1*β*- (IL-1*β*-) induced chondrocyte inflammation and apoptosis and may be a potential drug for the treatment of osteoarthritis [17]. Achyranthes bidentata polysaccharides (ABPS) can inhibit the expression of adhesion factors and chemokines, inhibit the activity of key enzymes in the development of inflammation, and regulate the production of cytokines and subsequently the adhesion and exudation of white blood cells to the site of inflammation [18]. ABPS can inhibit RANKL-induced osteoclast differentiation and bone resorption activity [19]. Additionally, ABPS can promote the proliferation of chondrocytes by activating the Wnt/*β*-catenin signaling pathway [19].

Coicis Semen is a dried and mature seed of Coix lacryma-jobi L.var.mayuen (Roman.) Stapf. Coicis Semen is not only a traditional Chinese medicine but also a kind of food. The main active components of Coicis Semen include fatty acids, lipids, sugars, sterols, alkaloids, and triterpenes. Modern pharmacological studies have shown that Coicis Semen has anti-inflammatory, analgesic, antitumor, and immune-enhancing properties among other pharmacological activities [20]. Adlay seed extract plays an anti-RA role by inhibiting proinflammatory factors and reducing oxidative stress, which may be achieved by inhibiting the mRNA expression of COX-2 and CHI3L1 in the ankle of RA rats and increasing the mRNA expression of CAT and GPx-1 [21]. Research on Coicis Semen has mostly focused on its antitumor effect. The antitumor mechanism of Coicis Semen has been reported to include inhibition of tumor cell division and proliferation, inhibition of tumor cell metastasis, induction of tumor cell apoptosis, and inhibition of tumor angiogenesis [22–24]. Injection of kanglaite, an antitumor agent extracted from Coicis Semen, inhibited the metastasis of colon cancer by inhibiting the epithelial-mesenchymal transformation induced by NF-*κ*B.

Simiao powder has been used to treat arthritis for hundreds of years. During this period, although some of its mechanisms have characterized, those underlying its role in KOA are lacking. CHMs have complex characteristics of multicomponent, multitarget, and multichannel activity. A single herb contains multiple medicinal active substances, which is further complicated by the use of multiple herbs in combination. The therapeutic effect of CHMs is established on the basis of the theory of synergy, and CHM compounds play an important role in the treatment of various diseases. However, the active components, targets, and mechanisms of CHM compounds in the treatment of diseases are not yet clear.

In 2007, Yildirim et al. conducted an analysis based on drug-gene and drug-protein interaction data sets and found that the drug and target tended to form an enriched network rather than an isolated corresponding relationship [25]. Moreover, most drug targets are related to many diseases, and a complex cross network is formed between drugs and disease genes. At the same time, Hopkins proposed the network pharmacology research method, believing that drugs act on multiple targets and produce effects through the interactions between multiple targets [26]. As an emerging technology field arising from fusion of multiple disciplines, network pharmacology aims to construct multilevel networks of various omics data analysis and computer simulation to understand the laws and mechanisms of the interactions between drugs and the body, providing an approach for systematic research on the complex system of traditional Chinese medicine [27].

**Figure 1:**
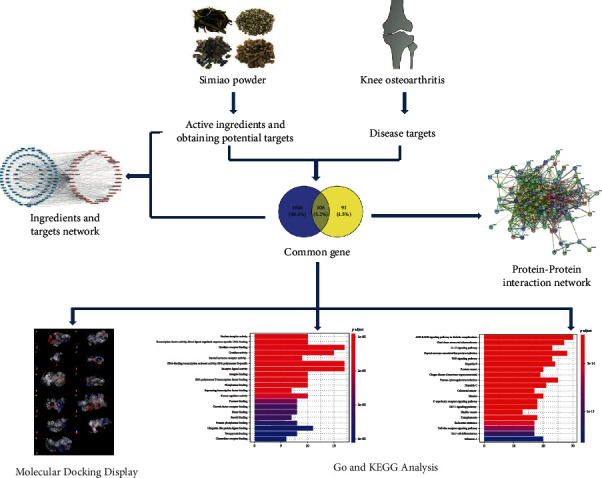
Whole framework based on network pharmacology.

Therefore, in this study, the mechanism of action of Simiao powder in the treatment of KOA was characterized by using network pharmacology and molecular docking technology, and the scientific basis was provided. This paper attempts to elucidate the specific targets and molecular signaling pathways in KOA influenced by Simiao powder and provide new strategies for new drug development and clinical use. The flow chart of this work is shown in Figure 1.

## 2. Materials and Methods

### 2.1. Inquiry and Screening of Gene Targets of Chemical Components of Simiao Powder

The Traditional Chinese Medicine Systems Pharmacology Database and Analysis Platform (TCMSP) is a unique tool for systematic pharmacology research of traditional Chinese medicines (TCMs) [28]. In TCMSP, the TCMs contained in Simiao powder were searched, including “Atractylodes chinensis rhizome,” “Cortex phellodendri,” “Radix Achyranthis Bidentatae,” and “Coicis Semen.” The potential targets of Simiao powder were collected. The screening conditions were as follows: oral bioavailability (OB) ≥ 30% and drug − likeness (DL) ≥ 0.18 [29, 30]. The UniProt database (https://www.UniProt.org/UniProt/) was used to determine the human gene abbreviation of each target and obtain the possible target proteins of Simiao powder.

### 2.2. Query and Prediction of KOA-Related Targets

“Knee osteoarthritis” was used as the search term in the OMIM database (https://www.OMIM.org/), GeneCard database (https://www.genecards.org/), DisGeNET database (https://www.disgenet.org), DrugBank database (https://www.DrugBank.ca), PharmGkb database (https://www.PharmGkb.org/), and Therapeutic Target Database (http://bidd.nus.edu.sg/bidd-databases/TTD/TTD.asp). The database search results were merged, and duplicate targets were deleted to obtain all targets of KOA. The intersection of the targeted prediction results of the active components of Simiao powder and the retrieval results of KOA-related targets was determined, and the common target was selected as the potential therapeutic target of Simiao powder in the treatment of KOA. The Venny 2.1 (http://bioinfogp.cnb.csic.es/tools/venny/index.html) online tool was used to map the active ingredient target and the disease target of Simiao powder and to draw Venn diagrams. Then, the cross targets of Simiao powder and KOA were collected as the common genes of RAB in KOA treatment, which may be the potential target sets of Simiao powder in KOA treatment.

### 2.3. Construction of Drug Active Ingredient and Disease Target Network

The screening results of the active components of Simiao powder and KOA targets were introduced into Cytoscape to construct the active components and KOA target network of Simiao powder and to visualize the network. Cytoscape is an open-source software platform for visualizing complex networks and integrating them with any type of attribute data [31]. The core architecture of the Cytoscape software is the network. Each node is a protein, gene, or active ingredient, and the connection is the interaction between these biomolecules. However, the interaction between nodes in these networks—which may be activation, inhibition, binding, catalysis, and other types of action as well as positive, negative, and nonspecific effects—remains unclear, which is a limitation of this approach.

### 2.4. Network Topological Feature Set Definition

For each node in the interactive network, we chose three parameters to evaluate its topological characteristics, which were obtained through CytoNCA. In this study, degree centrality (DC) is the number of connections between a node and other nodes in a network, and it is the most direct metric to depict the centrality of nodes in network analysis. The larger the degree, the more important the node [32]. Betweenness centrality (BC) is the ratio of the number of times a node takes the shortest path between the other two nodes to the total number of shortest paths. The more times a node serves as an intermediary, the greater the value and the more influence it has in the network [32, 33]. Closeness centrality (CC) reflects the closeness between a node and other nodes in the network [34]. The reciprocal of the sum of the shortest path distances of a node to all other nodes represents the proximity centrality. That is, the closer a node is to other nodes, the more central its proximity is. The greater the value of DC, BC, and CC, the more important the role of the node in the network.

### 2.5. Protein-Protein Interaction (PPI) Network Construction and Core Gene Screening

PPI is the basis of cell function of the body and plays an important role in regulating physiological and pathological states of the body. A protein-interaction network (PIN) is a network constructed with protein-interaction information. PIN uses network nodes to represent proteins, network connecting lines to represent protein interactions, and node size, color, connection length, and thickness to represent topology parameters of node network. PPI and access networks are important in most biological functions and processes. The STRING database (https://STRING-db.org/) is a database for searching known protein interactions and predicting protein interactions. By collecting, evaluating, and integrating all common “protein-protein” interaction resources, it complements the results predicted by the computer. On this basis, the common gene targets of Simiao powder and KOA were imported into the STRING database. A confidence level of 0.7 was selected, and the node on which the network was interrupted was hidden to analyze the protein-interaction network. The analysis results are saved in TSV format and imported into Cytoscape for topology attribute analysis. A node with high DC, BC, and CC values plays a very important role in the network. According to the analysis results of the topological properties of PPI mentioned above, the target with a median value above is selected as the core target, and the target is screened twice. Based on the results of the methods described above, we identify the hub gene of the network.

### 2.6. Enrichment Analysis of GO and KEGG

Bioconductor is a common open-source toolkit for analyzing and understanding high-throughput genomic data. Based on the R software, the tool provides a variety of analysis and visualization methods for high-throughput genomic data, as well as GO annotation information of 19 species including human. In this study, the target name of Simiao powder intervention in KOA was transformed into an Entrez ID using Perl language tools. Then, we used the GO annotation information provided by Bioconductor and KEGG pathway database (https://www.genome.jp/kegg/pathway.html, Last updated: March 10, 2020) for signaling pathway enrichment analysis; its visual function features are powerful. On the one hand, GO functional enrichment analysis includes categorization of molecular function (MF), biological process (BP), and cellular components (CC). On the other hand, KEGG enrichment analysis is used to screen the KEGG pathways. Using the above tools, we set the *p* value cutoff to 0.05 and *q* value cutoff to 0.05, selected the top 20 items with the highest enrichment, and displayed them in a bar chart. We downloaded 20 access maps and selected the possibly related ones after screening.

### 2.7. Molecular Docking

Discovery Studio 2016 3.0 is a new generation of a molecular modeling and simulation environment that is applied to protein structure and function research and drug discovery [16]. We used this software to conduct molecular docking and explain how ligands act on complex molecular networks. In this study, all the hub genes and the main effective components of Simiao powder were verified by molecular docking. A LibDock score was generated if the molecule successfully docked with the target protein, while the LibDock score of the ligand provides a baseline value defining the affinity between the protein and the molecule [35]. To evaluate the binding activity between the active components and the target of Simiao powder, the results of molecular docking were preserved and LibDock score was analyzed.

Through construction of the network of active components and disease targets, the drug-like components and KOA targets of Simiao powder were obtained. The mol2 structure of the pharmaceutical active ingredient was obtained from the Chemical Book database (https://www.chemicalbook.com/). The protein crystal structure of KOA-related target proteins was obtained from the RCSB PDB database (https://www.rcsb.org/).

The results obtained above were imported into the Discovery Studio software for initial processing. For active drug molecules, Prepare Ligands was used with parameters set to default values. For the target protein, the processing steps are as follows: prepare protein>automatic preparation>prepare protein. Through the above steps, the protein preparation process of hydrogenation, addition of missing atoms, and adjustment of protonation state was automatically completed. The specific binding site in the target protein was defined according to the crystal structure of the protein-ligand compound. The LibDock module docking conditions were as follows: docking preferences were set to high quality, conformation method was set to FAST, parallel processing was set to true, and other parameters were set to default values. The higher the LibDock score, the higher the predicted activity of target binding.

## 3. Result

### 3.1. Screening the Active Ingredients of Simiao Powder

We searched the TCMSP database, screened with OB ≥ 30% and DL ≥ 0.18, retrieved a total of 75 active ingredients in Simiao powder, removed 38 unrelated active ingredients, and finally obtained a list of 37 effective active ingredients (Supplementary Table 2).

### 3.2. Construction of the Effective Active Ingredient Library of Simiao Powder and the Target Set of KOA

The component targets from the TCMSP database contained a total of 1070 target targets for Simiao powder. We used the UniProt database to collect the gene name of the targets, removed the invalid and duplicate targets, and finally obtained 197 active ingredients. Through the search and integration of major databases, 1952 KOA-related gene targets were obtained (Supplementary Table 1). We mapped the 197 compound targets with 1952 KOA-related target genes to obtain 106 common target genes (Supplementary Table 3).

### 3.3. Drug Active Ingredient and Disease Target Network

**Figure 2:**
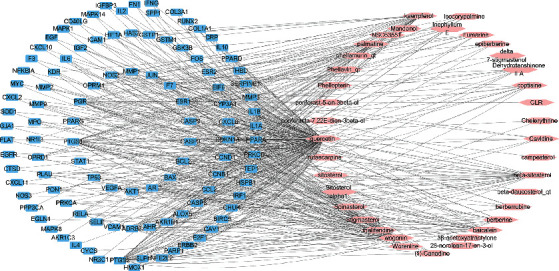
Drug active ingredients and disease target network. The pink ellipse represents the active ingredient, and the blue quadrilateral represents the target of drug action.

We used the Simiao powder therapeutic targets and corresponding active ingredients to build a network and imported it into Cytoscape for visualization, as shown in Figure 2. The network has 143 nodes (37 active ingredients and 106 therapeutic targets) and 455 edges. The ellipse represents the active ingredient, and the four rows represent the targets of drug action. One active ingredient corresponds to different targets, indicating that the active ingredients of Simiao powder can act on different targets in the network at the same time and coordinate the protein-interaction network. The topological analysis results of ingredient-target pair information are shown in Supplementary Table 4.

### 3.4. Interaction Network

**Figure 3:**
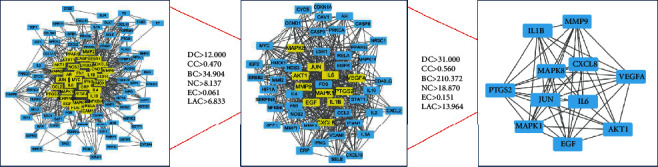
Process of topological screening for the PPI network.

We uploaded the common genes to the STRING database to build a protein-interaction network to obtain their interactions. Then, we imported the TSV data into Cytoscape for visualization. We used CytoNCA to calculate the topological parameters in the network nodes to obtain the DC, BC, CC, NC, EC, and LAC parameter values. The threshold for the first screening was DC > 12.000, CC > 0.470, BC > 34.904, NC > 8.137, EC > 0.061, and LAC > 6.833; the results show that there were 54 nodes and 583 edges in total. The second screening threshold was DC > 31.000, CC > 0.560, BC > 210.372, NC > 18.870, EC > 0.151, and LAC > 13.964. The second screening result was 11 nodes and 52 edges, including IL1B, MMP9, CXCL8, MAPK8, JUN, IL6, MAPK1, EGF, VEGFA, AKT1, and PTGS2 (Supplementary Table 5 and Figure 3).

### 3.5. Enrichment Analysis of GO and KEGG Channels

**Figure 4:**
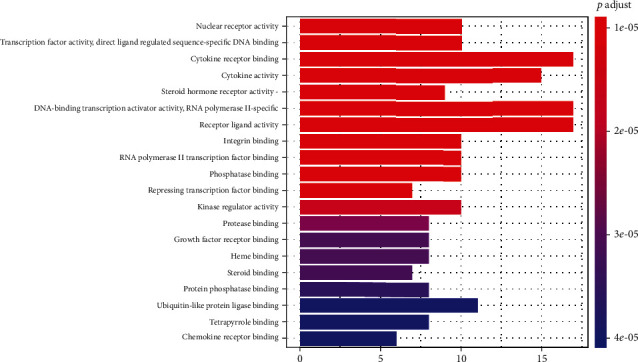
GO enrichment of Simiao powder active components in the treatment of common targets of knee osteoarthritis.

**Figure 5:**
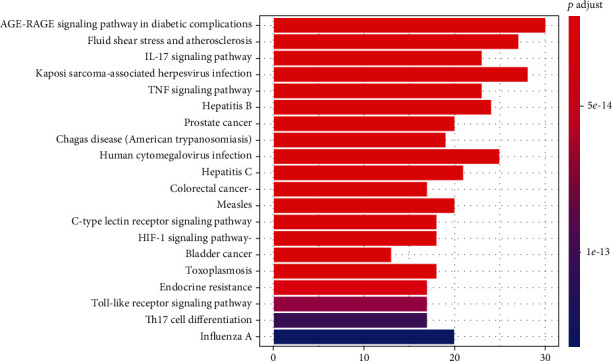
Enriched KEGG pathways of potential targets for treating knee osteoarthritis from the main active ingredients of Simiao powder.

GO enrichment analysis: we used clusterProfiler to determine the enrichment of the GO's biological process (BP), molecular function (MF), and cellular component (CC). The results showed that the targets of Simiao powder for KOA were enriched for 123 GO terms. We screened the top 20 GO analysis results with *p* < 0.05 as the threshold (Figure 4). KEGG enrichment analysis: to clarify the role of the Simiao powder treatment targets in signaling pathways, clusterProfiler was used to perform KEGG pathway enrichment analysis with *p*valueCutoff = 0.05. A total of 148 pathways were enriched, and the top 20 pathways are shown in Figure 5. These pathways are related to the AGE-RAGE signaling pathway in diabetic complications, IL-17 signaling pathway, TNF signaling pathway, Toll-like receptor signaling pathway, and HIF-1 signaling pathway, suggesting that the pathways targeted by Simiao powder may reduce inflammation and inhibit infection in the treatment of KOA.

### 3.6. Target Path Analysis

**Figure 6:**
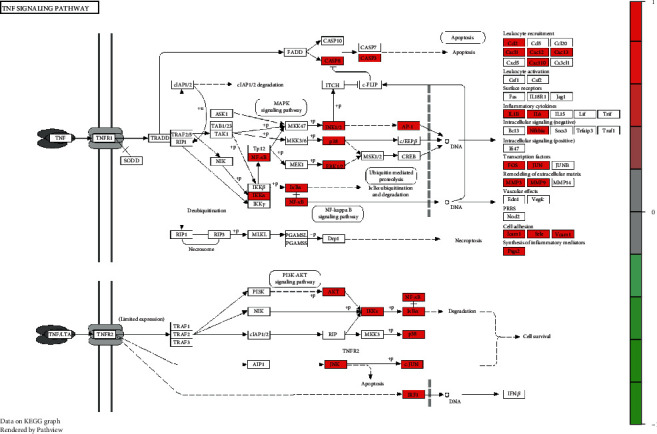
Pathway map of Simiao powder in the treatment of knee osteoarthritis. The main targets of Simiao powder in the treatment of knee osteoarthritis are located in the apoptosis pathway. Arrows represent the activation effect, T arrows represent the inhibition effect, and segments show the activation effect or inhibition effect.

The KEGG Mapper tool was used to obtain the pathway map of Simiao powder for the treatment of KOA, as shown in Figure 6. The pathway targets were marked in white, and the targets of Simiao powder for the treatment of KOA were marked in red. The pathway map showed the pathways influenced by Simiao powder in the treatment of KOA, including the TNF signaling pathway, IL-17 signaling pathway, HIF-1 signaling pathway, and Toll-like receptor signaling pathway. For example, in the TNF signaling pathway, Simiao powder has accumulated 34 targets in the treatment of KOA. It was suggested that Simiao powder may play a role in the treatment of KOA by regulating several aspects, and its targets may be located in these pathways.

### 3.7. Molecular Docking Analysis

**Figure 7:**
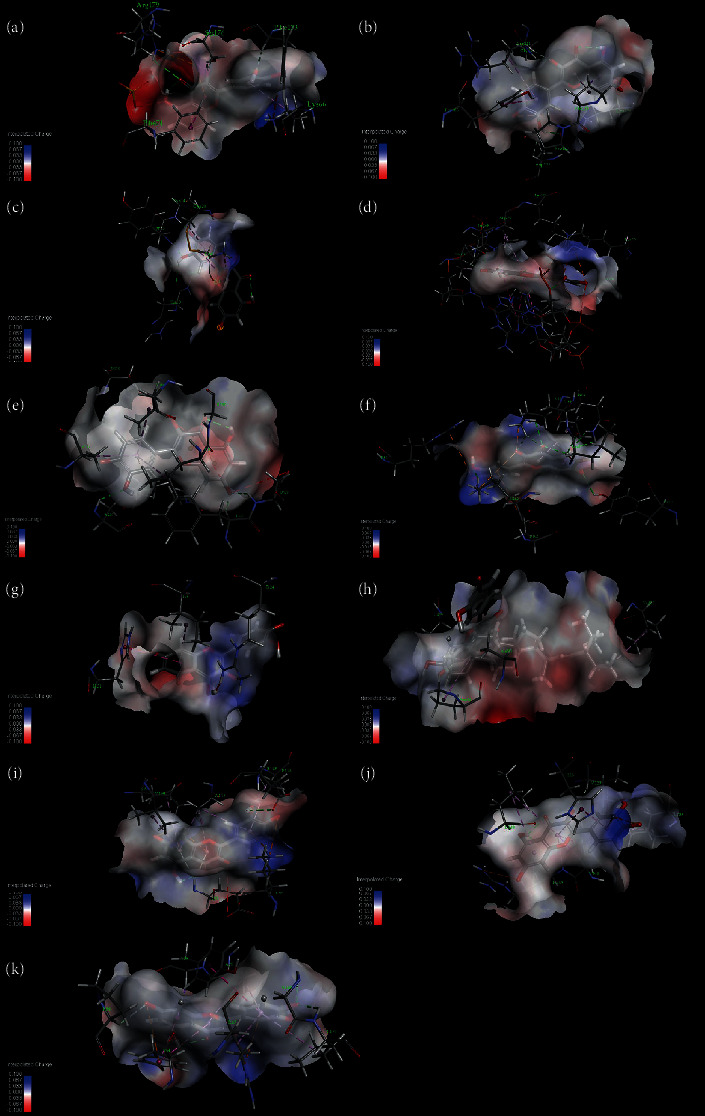
Molecular docking of compounds with core targets. (a) Docking process of quercetin with IL6; (b) docking process of quercetin with VEGFA; (c) docking process of quercetin with EGF; (d) docking process of quercetin with JUN; (e) docking process of quercetin with AKT1; (f) docking process of quercetin with MAPK8; (g) docking process of quercetin with CXCL8; (h) docking process of quercetin with PTGS2; (i) docking process of quercetin with IL1B; (j) docking process of quercetin with MMP9; (k) docking process of quercetin with MAPK1.

The results of molecular docking show that the active ingredients of Simiao powder match well with the hub genes. The mechanism of Simiao powder's treatment of KOA was partially explained by the regulation of the targets of IL1B, MMP9, CXCL8, MAPK8, JUN, IL6, MAPK1, EGF, VEGFA, AKT1, and PTGS2. This indicates that the molecular docking results are consistent with the results of network pharmacology screening, and the reliability of network pharmacology is verified by molecular docking. The docking results for Simiao powder's active ligand and KOA target protein receptor are shown in Supplementary Table 6 and Figure 7.

## 4. Discussion

Some previous studies have shown that Simiao powder inhibits inflammation. Simiao powder can inhibit the production of proinflammatory cytokines and reduce cartilage and bone injury by regulating the ATX-LPA and MAPK pathways [36]. A rabbit KOA model shows that Simiao powder can inhibit the levels of IL-1*β* and TNF-*α*, inhibit the inflammatory reaction effectively, downregulate the expression of IL-6, and upregulate the expression of basic fibroblast growth factor (bFGF), to reduce the degradation of cartilage matrix [37, 38]. Additionally, it is suggested that Simiao powder may play a positive role in the regulation of chondrocyte apoptosis and autophagy in rats with KOA, thus delaying the pathological process of KOA [39]. Although some studies on the mechanism of Simiao powder in the treatment of KOA have been carried out, our understanding is insufficient, and there is still a need for exploration in this field.

Based on the network pharmacology approach, 37 active ingredients and 106 potential targets of Simiao powder were screened by using the target database of traditional Chinese medicine compounds and a human disease gene database. The enrichment of GO and KEGG terms in the targets of Simiao powder acting on KOA was analyzed with several biological function databases. Quercetin, wogonin, kaempferol, beta-sitosterol, and other active ingredients play an important role in the treatment of KOA with Simiao powder. The degree value of quercetin is 174, which is much higher than that of other compounds.

Quercetin is a flavonol compound with many biological activities; it is widely distributed in the plant kingdom and has many functions such as antioxidation, antiviral, and anti-inflammatory effects. Quercetin can inhibit the apoptosis of chondrocytes in the knee joint, reduce the production of proinflammatory cytokines, weaken the oxidative stress response, inhibit the degradation of cartilage extracellular matrix, protect articular cartilage, and delay the progression of OA [40]. Wogonin can activate the ROS/ERK/Nrf2 signaling pathway in OA chondrocytes, and thus, it plays an anti-inflammatory and protective role in cartilage [41]. Kaempferol is a flavonoid chemical with various biological functions such as anti-inflammatory, analgesic, and anticancer effects. Kaempferol can reduce the expression of iNOS and COX-2 and reduce the interleukin-1*β*-induced inflammation of rat OA chondrocytes by inhibiting NF-*κ*B, thereby protecting OA rat chondrocytes [42]. Another study shows that kaempferol may inhibit IL-1*β*-stimulated inflammation by inhibiting the MAPK pathway [43]. Beta-sitosterol is a common phytosterol, which is widely found in plants. Related studies have shown that beta-sitosterol effectively inhibits the generation of reactive oxygen species (ROS) induced by LPS, partially inhibits the activation of NF-*κ*B, and significantly inhibits NLRP3 inflammatory bodies. Additionally, beta-sitosterol also regulates bone metabolism balance and antioxidation [44].

Through topology analysis of the PPI network, 11 hub genes were identified including IL1B, MMP9, CXCL8, MAPK8, JUN, IL6, MAPK1, EGF, VEGFA, AKT1, and PTGS2.

IL6 is a mediator of inflammation and immune response, can be detected in synovial fluid, and is expressed in osteoarthritis cartilage, making IL-6 inhibition a potential target for the treatment of KOA [45, 46]. Activator protein-1 (AP-1) is a homodimer and heterodimer composed of JUN and FOS family members, and it is a oncogene or tumor suppressor [47]. Some studies have shown that the MMP promoter contains a potential AP-1 binding site, and IL-1*β* can also activate AP-1-mediated transcription in articular chondrocytes. IL-1*β* induces MMP13 and inhibits bone and joint by inhibiting BATF/JUN transcription activity and inflammation of cartilage [48–50]. AKT is a serine/threonine protein kinase and an important downstream target kinase in the PI3K signaling pathway; AKT1 is one of its subtypes. Activated AKT affects the activation of downstream effector molecules. The PI3K/AKT signal transduction pathway is an important pathway that works against chondrocyte apoptosis [51]. VEGFA participates in a variety of pathological reactions of OA, including cartilage degeneration, osteophyte formation, synovitis, and pain, and is positively correlated with OA progression [52]. It can reduce OA progression by inhibiting VEGF signaling [53]. Chemokines are secreted small molecules, and research shows that CXCL8 may promote chondrocyte apoptosis and inhibit chondrocyte proliferation [54]. Matrix metalloproteinases (MMPs) play an important role in bone development and bone reconstruction, and they have several common structural features, such as the presence of a conserved zinc-binding catalytic domain. MMP9 is widely expressed in noninfected normal connective tissues; it participates in the degradation of extracellular matrix, inflammatory response, and immune response and is related to the pathogenesis of osteoarthritis [55]. A genetic polymorphism study showed that the incidence of OA was significantly higher in the IL1B T-31C T/T genotype compared to the C/C and C/T genotypes [56]. However, whether IL1B is related to OA is unclear [57]. PTGS2 is a prostaglandin-endoperoxide synthase and an effective mediator of inflammation, and it can produce inflammatory prostaglandins [58]. Epidermal growth factor (EGF) can activate the EGFR pathway, and this signaling plays a vital role in cartilage development and homeostasis [59].

The enrichment analysis showed that Simiao powder may play a role in the prevention and treatment of KOA by regulating the AGE-RAGE in diabetic complications, IL-17, TNF, Toll-like receptor, and HIF-1 signaling pathways. In the process of aging, advanced glycation end products (AGEs) accumulate in articular cartilage.

The biological activity of AGEs is believed to be mediated by the age-specific receptor (RAGE), and activation of RAGE is involved in key signaling pathways related to activation of the proinflammatory response and various inflammatory genes [60]. On the one hand, AGEs induce inflammation by activating NF-*κ*B and MAPKs in various cell types, including chondrocytes [61]. On the other hand, AGEs may increase the levels of PGE (2) and NO through the MAPK pathway to enhance the inflammatory response of OA chondrocytes [62]. Additionally, AGEs stimulate endoplasmic reticulum stress of human chondrocytes through RAGE, resulting in the phosphorylation of eIF2*α* and p38 MAP kinases and the activation of NF-*κ*B, which in turn increases COX-2 expression and PGE2 production [61]. IL-17 is one of many inflammatory cytokines and is mainly produced by T cells. The cells affected by IL-17 in joints are mainly chondrocytes. Imaging studies showed that images of OA lesions were positively correlated with increased IL-17 levels in serum and synovial fluid [63]. Additionally, IL-17 can induce chondrocytes and synovium fibroblasts to release chemokines and induce chondrocytes to synthesize IL-1, thereby promoting the pathological progression of OA [64].

The TNF signaling pathway, especially TNF-*α*, is known to play an important role in the pathological progress of OA. TNF-*α* can bind to two kinds of membrane receptors: TNF-R1 and TNF-R2. TNF-R1 can be activated in its soluble and membrane form, while TNF-R2 is mainly bound as its membrane form [65]. Compared with TNF-R2, TNF-R1 causes more damage to articular cartilage; however, both of them play an important role in the development of OA [66]. The binding complexes further activate the transcription factor NF-*κ*B and the MAPK signaling pathway. The activation of various signaling pathways can induce the production of matrix metalloproteinases, including MMP1, MMP3, and MMP13, which can enhance inflammation and further aggravate the destruction of OA cartilage [67].

Toll-like receptors (TLRs) are transmembrane signal transduction receptors. As a type I transmembrane protein, TLRs can be divided into three parts: extracellular, cytoplasmic, and transmembrane regions [68]. TLRs are expressed in articular cartilage and are upregulated in OA cartilage. TLR expression and signal transduction are related to the pathogenesis of OA [69].

TLR1-7 and TLR9 are upregulated in the synovium of patients with OA, and TLR4 is related to the severity of OA [70]. Activation of TLRs and subsequent activation of NF-*κ*B stimulate the release of IFN regulatory factors and the MAPK signaling pathway, leading to the release of chemokines, proinflammatory factors, and IFN. The TLR2/TLR4 signaling pathway induces expression of IL-1 and MMPs, increases the production of NO and PGE2, accelerates the degradation of proteoglycan and type II collagen, leads to the destruction of articular cartilage, and promotes the formation of KOA [71]. Additionally, TLR4 expression seems to be regulated by the shear stress of chondrocytes. High shear stress leads to upregulation of TLR4, and long-term shear stress leads to downregulation of TLR4 and TLR4-dependent inflammatory responses [72].

Hypoxia inducible factors (HIFs) are key regulators of transcription factors and cell responses to hypoxia. So far, three members of the human HIF-*α* protein family have been described: HIF-1*α*, HIF-2*α*, and HIF-3*α* [73]. HIF-1 is an important factor that maintains chondrocyte homeostasis and allows cell differentiation [74]. HIF-1 is a heterodimer which consists of two subunits: HIF-1*α* and HIF-1*β*. One study shows that HIF-1*α* plays an important role in preventing OA. HIF-1*α* downregulates MMP13 by inhibiting *β*-catenin transcriptional activity in chondrocytes, thus alleviating the development of OA; lack of HIF-1*α* intensifies cartilage catabolism [75]. Additionally, HIF-1 is involved in the process of KOA synovium fibrosis [76].

Using network pharmacology, 37 active components and 1070 potential therapeutic targets of Simiao powder were explored in this study. There were 1952 KOA-related targets, 106 of which are shared between Simiao powder and KOA.

The GO biological process and KEGG signaling pathway enrichment analysis preliminarily predicted that Simiao powder regulates IL1B, MMP9, CXCL8, MAPK8, Jun, IL6, MAPK1, EGF, VEGFA, AKT1, PTGS2, and other targets through quercetin, wogonin, kaempferol, beta-sitosterol, and other effective active components; it regulates the AGE-RAGE signaling pathway in diabetic complications, IL-17 signaling pathway, TNF signaling pathway, Toll-like receptor signaling pathway, and HIF-1 signaling pathway and can inhibit inflammatory reaction, regulate immune function, and treat KOA.

Unfortunately, this study provided only preliminary predictions, and the results have not been verified in clinical and animal experiments. Limited by the deficiencies of systems biology, multidirectional pharmacology, computational biology, and network analysis, the conclusions still need to be verified with subsequent experiments. Although there are some limitations, the network pharmacology analysis results show that there are many and complex factors that cause KOA. The analysis also intuitively shows the characteristics of Simiao powder in the treatment of KOA. Such research will undoubtedly increase our understanding of the pathogenesis of KOA and lead to new treatment strategies in the prevention and treatment of KOA.

## Data Availability

The datasets used and/or analyzed during the current study are available from the corresponding author upon reasonable request.

## Abbreviations

CHM: Chinese herbal medicine
KOA: Knee osteoarthritis
OB: Oral bioavailability
DL: Drug-likeness
KEGG: Kyoto Encyclopedia of Genes and Genomes
PDB ID: Protein Data Bank identification
PPI network: Protein-protein interaction network
GO: Gene Ontology
ATL-1: Atractylenolide I
ABPS: Achyranthes bidentata polysaccharides
TCMSP: Traditional Chinese Medicine Systems Pharmacology Database and Analysis Platform
DC: Degree centrality
BC: Betweenness centrality
CC: Closeness centrality
PPI: Protein-protein interaction.

## Supplementary Materials

Supplementary Materials Supplementary Table 1: potential target information of Simiao powder for KOA. Supplementary Table 2: active ingredient parameters of Simiao powder. Supplementary Table 3: the common gene targets of Simiao powder in the treatment of KOA. Supplementary Table 4: compound-target pair information. Supplementary Table 5: topology parameters in the PPI network. Supplementary Table 6: results of ligand-receptor protein molecular docking.
