# Supplementary material for: Network Pharmacology Approach to Uncover the Mechanism Governing the Effect of Simiao Powder on Knee Osteoarthritis
Source: Biomed Res Int. 2020 Dec 7;2020:6971503. doi: 10.1155/2020/6971503 (PMC7738782; doi:10.1155/2020/6971503)
Supplement: Supplementary Materials — Supplementary Table 1: potential target information of Simiao powder for KOA. Supplementary Table 2: active ingredient parameters of Simiao powder. Supplementary Table 3: the common gene targets of Simiao powder in the treatment of KOA. Supplementary Table 4: compound-target pair information. Supplementary Table 5: topology parameters in the PPI network. Supplementary Table 6: results of ligand-receptor protein molecular docking. [file 6971503.f1.zip › Supplementary Tables/Supplementary Table 2 (1).docx]

| **MOL ID** | **Structure** | **Name** | **OB（%）** | **DL** |
| --- | --- | --- | --- | --- |
| MOL000173 | 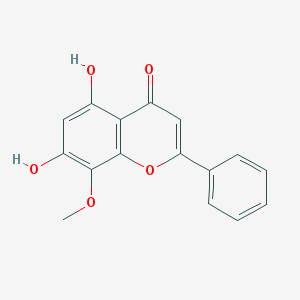 | wogonin | 30.68 | 0.79 |
| MOL000085 | 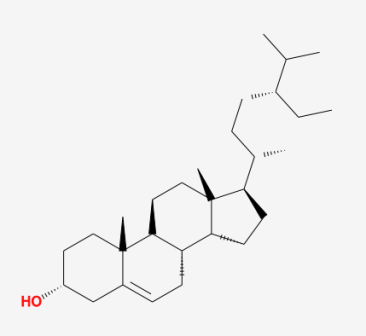 | beta-daucosterol_qt | 36.91 | 0.75 |
| MOL001454 | 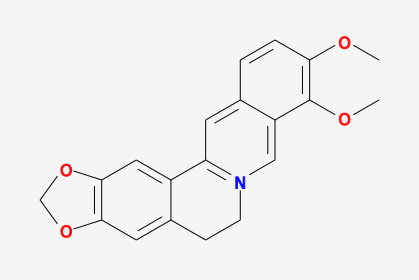 | berberine | 36.86 | 0.78 |
| MOL001458 | 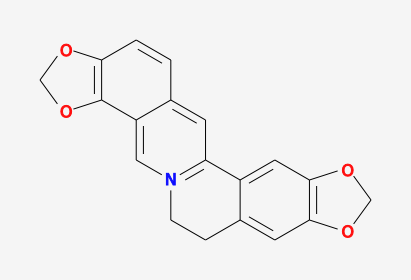 | coptisine | 30.67 | 0.86 |
| MOL002643 | 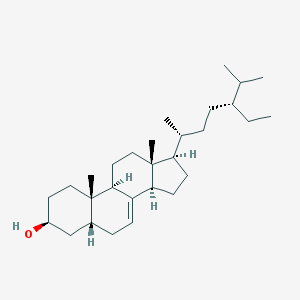 | delta 7-stigmastenol | 37.42 | 0.75 |
| MOL000449 | 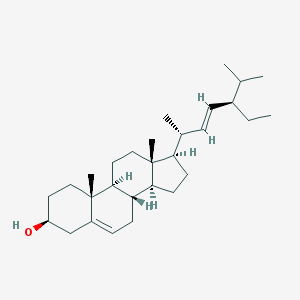 | Stigmasterol | 43.83 | 0.76 |
| MOL000358 | 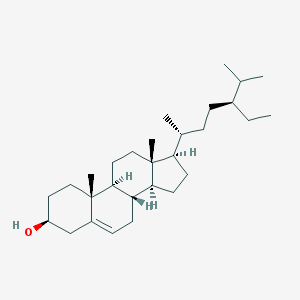 | beta-sitosterol | 36.91 | 0.75 |
| MOL000785 | 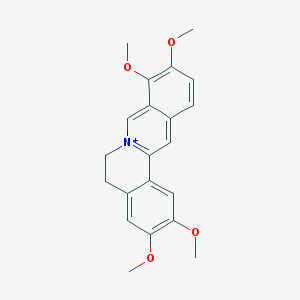 | palmatine | 64.60 | 0.65 |
| MOL000098 | 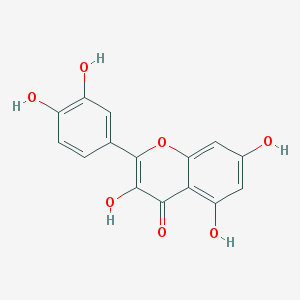 | quercetin | 46.43 | 0.28 |
| MOL001006 | 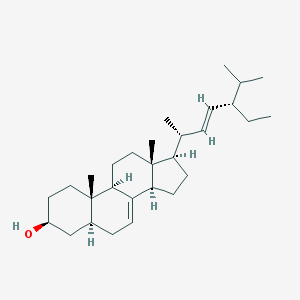 | poriferasta-7,22E-dien-3beta-ol | 42.98 | 0.76 |
| MOL012461 | 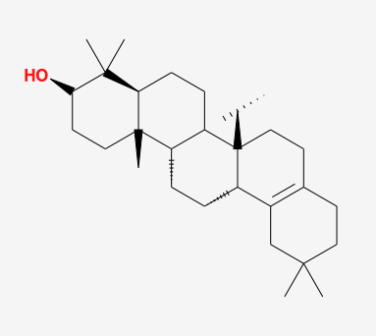 | 28-norolean-17-en-3-ol | 35.93 | 0.78 |
| MOL002714 | 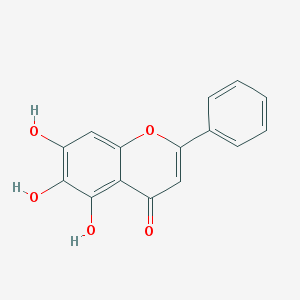 | baicalein | 33.52 | 0.21 |
| MOL002897 | 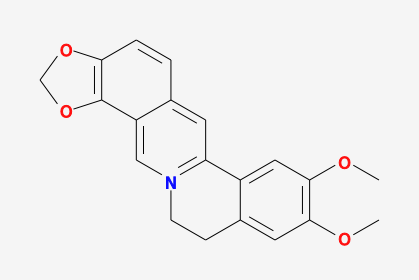 | epiberberine | 43.09 | 0.78 |
| MOL003847 | 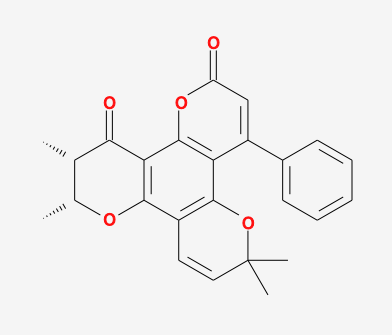 | Inophyllum E | 38.81 | 0.85 |
| MOL000422 | 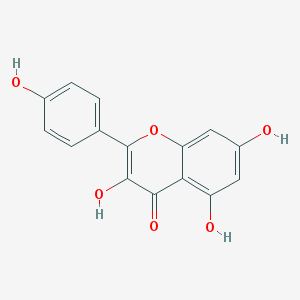 | kaempferol | 41.88 | 0.24 |
| MOL004355 | 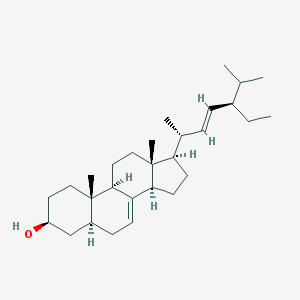 | Spinasterol | 42.98 | 0.76 |
| MOL000184 | 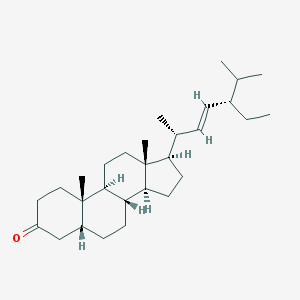 | NSC63551 | 39.25 | 0.76 |
| MOL000188 | 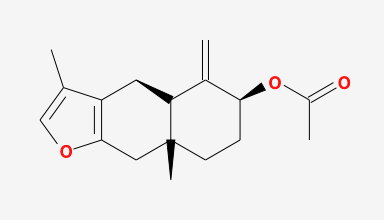 | 3β-acetoxyatractylone | 40.57 | 0.22 |
| MOL002641 | 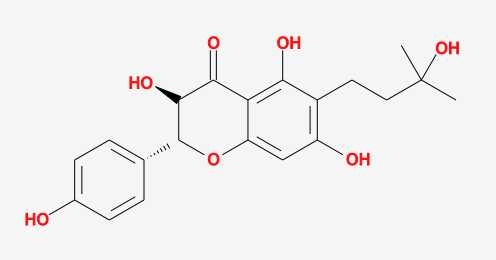 | Phellavin_qt | 35.86 | 0.44 |
| MOL002644 | 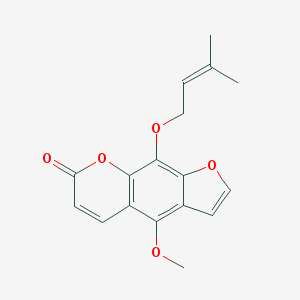 | Phellopterin | 40.19 | 0.28 |
| MOL002651 | 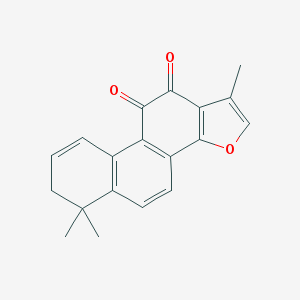 | Dehydrotanshinone II A | 43.76 | 0.40 |
| MOL002662 | 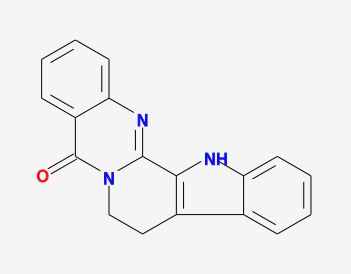 | rutaecarpine | 40.30 | 0.60 |
| MOL002666 | 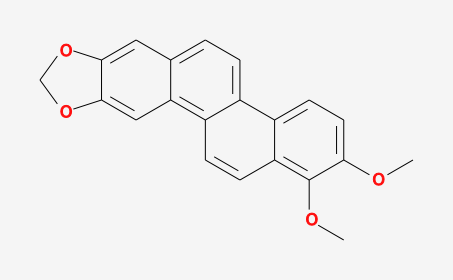 | Chelerythrine | 34.18 | 0.78 |
| MOL002668 | 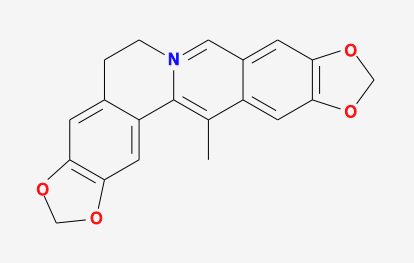 | Worenine | 45.83 | 0.87 |
| MOL002670 | 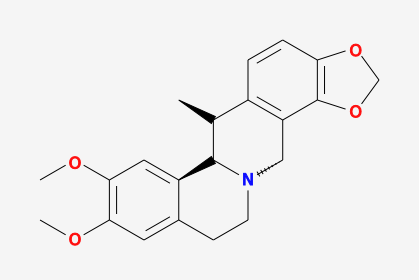 | Cavidine | 35.64 | 0.81 |
| MOL000787 | 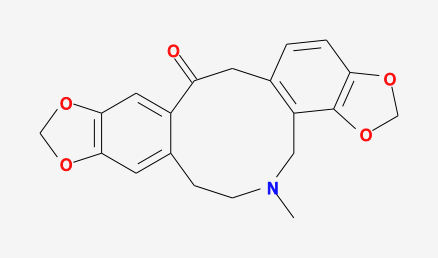 | Fumarine | 59.26 | 0.83 |
| MOL000790 | 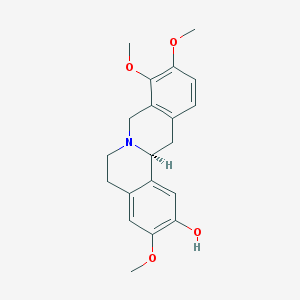 | Isocorypalmine | 35.77 | 0.59 |
| MOL001131 | 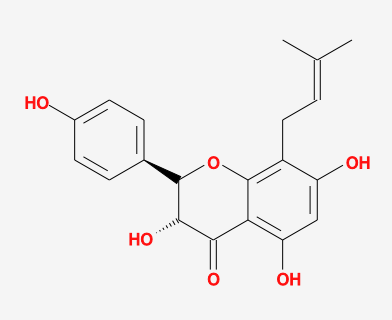 | phellamurin_qt | 56.60 | 0.39 |
| MOL001455 | 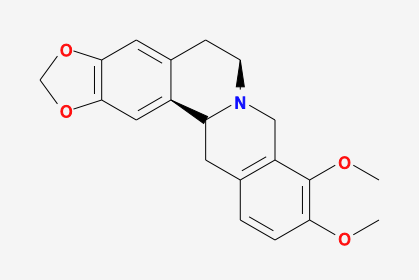 | (S)-Canadine | 53.83 | 0.77 |
| MOL001771 | 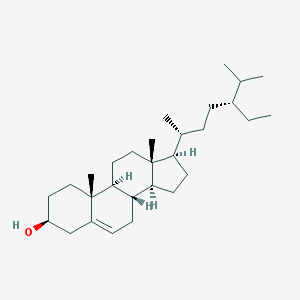 | poriferast-5-en-3beta-ol | 36.91 | 0.75 |
| MOL002894 | 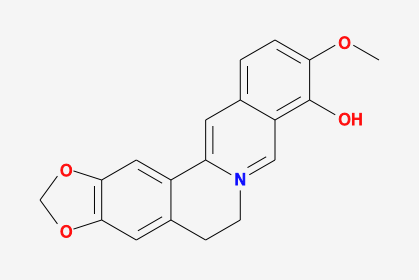 | berberrubine | 35.74 | 0.73 |
| MOL005438 | 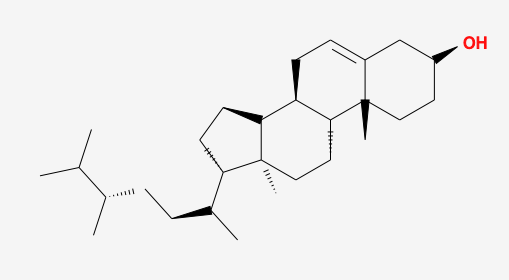 | campesterol | 37.58 | 0.71 |
| MOL006422 | 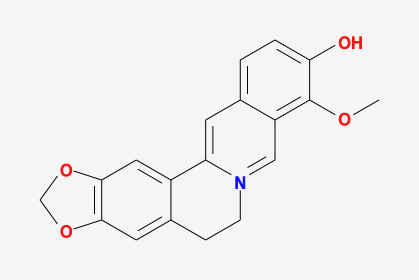 | thalifendine | 44.41 | 0.73 |
| MOL001323 | 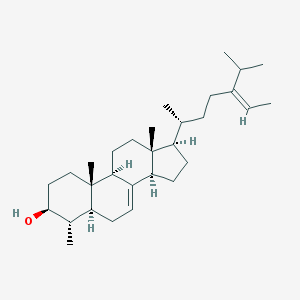 | Sitosterol alpha1 | 43.28 | 0.78 |
| MOL001494 | 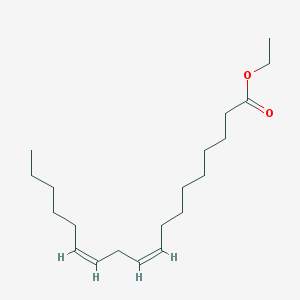 | Mandenol | 42.00 | 0.19 |
| MOL000359 | 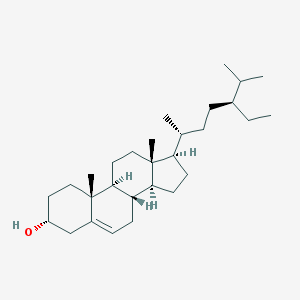 | sitosterol | 36.91 | 0.75 |
| MOL000953 | 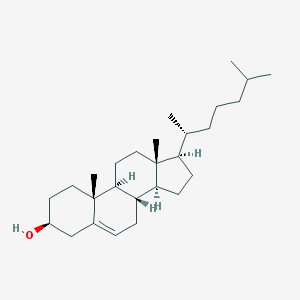 | CLR | 37.87 | 0.68 |

**Supplementary Table 2:** **Active ingredients parameters of Simiao Powder.**

* Oral Bioavailability: OB; Drug-Likeness: DL
